# Correction: Plasticizer and catalyst co-functionalized PEDOT:PSS enables stretchable electrochemical sensing of living cells

**DOI:** 10.1039/d1sc90245h

**Published:** 2021-11-25

**Authors:** Jing Yan, Yu Qin, Wen-Ting Fan, Wen-Tao Wu, Song-Wei Lv, Li-Ping Yan, Yan-Ling Liu, Wei-Hua Huang

**Affiliations:** College of Chemistry and Molecular Sciences, Wuhan University Wuhan 430072 China yanlingliu@whu.edu.cn whhuang@whu.edu.cn; School of Pharmacy, Changzhou University Changzhou 213164 China

## Abstract

Correction for ‘Plasticizer and catalyst co-functionalized PEDOT:PSS enables stretchable electrochemical sensing of living cells’ by Jing Yan *et al.*, *Chem. Sci.*, 2021, **12**, 14432–14440, DOI: 10.1039/d1sc04138j.

The authors regret that there was an error in the equation of the calibration curve of PPL/PDMS in Fig. 3. The correct version is shown below.

**Fig. 3:**
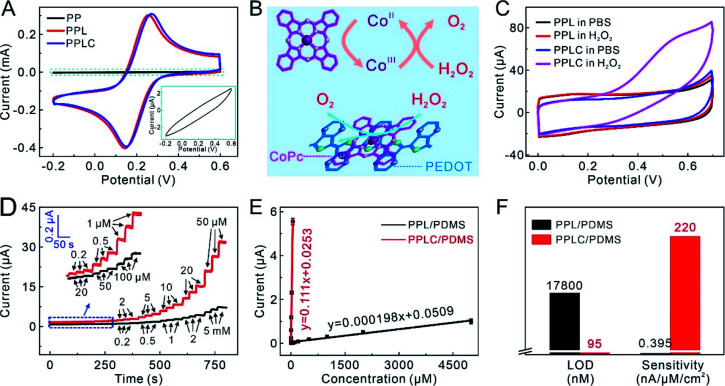
(A) CVs of different electrodes obtained in 10 mM K_3_[Fe(CN)_6_]. Inset: the enlarged view for CV of the PP electrode. (B) Schematic illustration of the electrocatalysis mechanism. (C) CVs of different electrodes with and without 1 mM H_2_O_2_. (D) Amperometric responses of PPL/PDMS (black lines) and PPLC/PDMS (red lines) electrodes to H_2_O_2_ at a potential of +0.55 V (*vs.* Ag/AgCl) to increasing H_2_O_2_ concentrations. Inset: the enlargements of amperometric responses framed in blue. (E) Calibration curves of PPL/PDMS and PPLC/PDMS electrodes to increasing H_2_O_2_ concentrations (data presented as mean ± standard error, *n* = 3). (F) Calculated LOD and sensitivity of PPL/PDMS and PPLC/PDMS electrodes to H_2_O_2_.

The Royal Society of Chemistry apologises for these errors and any consequent inconvenience to authors and readers.

## Supplementary Material

